# Social and environmental factors impacting the health in adults experiencing episodic homelessness in Houston, Texas

**DOI:** 10.1016/j.pmedr.2026.103681

**Published:** 2026-10-23

**Authors:** Neki Yazdi, Laura Porterfield, Namita Bhardwaj, Kendall M. Campbell, Truong Lam, Emily Rodgers, Geraldo Hernandez, Audrey Nath

**Affiliations:** a University of Pennsylvania, Philadelphia, PA, United States of America; b University of Texas Medical Branch, Galveston, TX, United States of America; c MD Anderson Cancer Center, Houston, TX, United States of America; d McGovern Medical School, Houston, TX, United States of America; e University of Houston-Clear Lake, Houston, TX, United States of America

**Keywords:** Social factors, Environmental factors, Health outcomes, Episodic homelessness, Houston

## Abstract

**Objective:**

While poorer health outcomes have been established in people experiencing episodic homelessness in the United States, there is a gap in understanding of the effects of other non-medical factors, such as employment, transportation access, and food insecurity, on health outcomes in this population.

**Methods:**

This was an observational study with data collected by in-person and phone interviews, involving 40 adults experiencing episodic homelessness in the greater Houston area from September 2024 through July 2025.

**Results:**

Lack of reliable food access showed a trend towards a significant association with self-described health status (*p* = 0.05), with participants with limited access to food more likely to report poor health (30.00%) than those with reliable food access (5.56%). Employment status showed a significant association with having reliable food access (*p* = 0.03), with participants with limited access to food more likely to report having no employment (85.71%) than those with reliable food access (52.94%). Transportation access and gross financial status did not show any association with food access.

**Conclusions:**

In individuals experiencing episodic homelessness in the Houston area, reliable food access had a trend towards a significant association with self-reported health status, and having employment was significantly associated with reliable food access.

## 1 Introduction

Housing insecurity is a well-established risk factor for poor medical outcomes and is associated with roughly double the mortality rate compared with housed populations (Morrison, 2009). In addition to higher rates of physical illness and mortality, unhoused populations also have higher rates of mental illness and substance use (Richards and Kuhn, 2022).

Unhoused populations are often categorized by chronicity of homelessness (Franco et al., 2021). Chronic homelessness is generally defined by longer durations of homelessness, typically at least 12 months, while episodic homelessness refers to repeated, short-term episodes, such as cycling between shelters, temporary housing, and periods of unstable housing (McAllister et al., 2011).

In the United States (U.S.), there are large-scale federal programs focused on ending chronic homelessness, including the Department of Housing and Urban Development (HUD) Homelessness Assistance Grants and the HUD and Veterans Affairs Supported Houston Program. While major research efforts and aid are devoted towards helping populations experiencing chronic homelessness, the number of people experiencing episodic homelessness may be overlooked. For instance, the main method for by HUD for tracking homelessness in the U.S. is with a single-night “point in time” (PIT) count, which may undercount the number of unhoused people due to lack of inclusion of people who may shuttle between staying with friends or family without their own housing (de Sousa and Henry, 2024).

Hence, while the population of people experiencing episodic homelessness may receive less of a focus from both funding bodies and researchers alike, there is importance in studying the needs and vulnerabilities of people experiencing episodic homelessness given that this population is at risk for chronic homelessness, as well as health disparities compared to the general population (Morrison, 2009).

Additionally, while there has been previous research into the relationship between episodic homelessness and various health outcomes, there is a gap in understanding of the effects of other non-medical factors that have been shown to affect health outcomes in populations experiencing homelessness, such as employment (Leddy et al., 2014), transportation access (Murphy, 2019), and food insecurity (Tucker et al., 2024).

A better understanding of how non-medical factors influence health outcomes among the episodically homeless population could support more effective policies, improve service delivery, advance theoretical knowledge and guide future research. Such knowledge would help shift attention from treating illness alone to addressing the broader social conditions that contribute to health disparities and recurring homelessness, leading to more sustainable improvements in both health and housing stability.

The purpose of this research is to explore whether non-medical factors in individuals who meet criteria for episodic homelessness, but not chronic homelessness, in the greater Houston area have an association with health status.

## 2 Methods

### 2.1 Study design and population

This study was approved by the University of Texas Medical Branch Institutional Review Board and complied with the Health Insurance Portability and Accountability Act.

Inclusion criteria were as follows: people aged 18 years and above who are experiencing episodic homelessness in greater Houston, Texas from September 2024 through July 2025. Episodic homelessness was defined as self-reported homelessness for under 12 months, with at least 3 interruptions in housing, or episodes of homelessness, within the past year. Participants were not included in this study if they did not speak English or Spanish, were under the age of 18, or were not episodically homeless.

This was an observational study of adults experiencing episodic homelessness in the greater Houston area, with data collected by in-person and phone interviews. Each participant was approached either in person or by telephone sequentially after being referred to a Houston-based non-profit organization that serves individuals experiencing episodic homelessness. Verbal consent was obtained and documented.

A target sample size of 61 was calculated based on an estimated effect size (w) of 0.36 based on previously published data from a similar population of individuals experiencing homelessness (Shadravan et al., 2020), 1 degree of freedom for a chi-squared comparison of proportions, significance level of 0.05 and power of 0.80 (Abdul Rahman et al., 2025).

### 2.2 Measures

Each participant completed a novel questionnaire which included demographics (age, sex, race/ethnicity), as well as social factors (food access, employment status, and access to transportation); the social factors questions are shown in Supplemental Fig. 1 (S1). Overall self-assessment of health condition was quantified with a 3-point Likert scale similar to that of a large population-based study of self-rated health outcomes (Wu et al., 2013).

### 2.3 Statistical analysis

Descriptive statistics were used on the collected categorical data (yes/no questions, Likert rating scales), to determine the percentage of subjects who had each response category. For continuous variables, such as age, descriptive statistics were performed. A chi-squared test was performed to determine if there was any association between the above-listed social risk factors and health condition self-report.

If there was any statistical significance (*p* < 0.05) or trend towards statistical significance between a social risk factor and self-reported health condition, further chi-squared analyses were performed between that social risk factor and the rest of the studied social risk factors, to determine if any of these risk factors had a statistically significant relationship. All statistics were calculated using Microsoft Excel (Microsoft Corporation, 2018, Microsoft Excel).

## 3 Results

### 3.1 Demographics

Forty participants met inclusion criteria for this study and consented to questionnaires. Average participant age was 47.08 years, and 52.50% were male. Sex was not significantly associated with health status (*p* = 0.79).

### 3.2 Data analysis and statistics

The response breakdowns for each studied social factor are listed in Table 1. Of note, the total numbers for each category reflect the number of participants who had complete data on all relevant study variables, and there were some questions that were not answered by some participants.

**Table 1:** Response breakdown to each of the studied social factors, by fraction. The numerators represent the number of adults experiencing episodic homelessness in Houston, Texas in the 2024–2025 timeframe who gave this particular response, and the denominators represent the total number of respondents to this social factor question.

Social factor	Prevalence	n (%)
Food	Inconsistent Reliable	20 / 38 (52.63%) 18 / 38 (47.37%)
Health	Poor Satisfactory	7 / 38 (18.42%) 31 / 38 (81.58%)
Employment	None Reliable income	27 / 38 (71.05%) 11 / 38 (28.95%)
Transportation	Inconsistent Reliable	30 / 38 (78.95%) 8 / 38 (21.05%)

Lack of reliable access to food was the only factor found to have a trend towards significant association with self-described health status (*p* = 0.05, chi-squared test; Table 2). Participants with inconsistent, temporary, or no access to food were more likely to report poor health (30.00%) than those with reliable food access (5.56%). The strength of the association was small-to-moderate (φ = 0.32).

**Table 2:** Chi-square tests were conducted to examine associations between food access and health status (A) and employment status (B). Adults experiencing episodic homelessness in Houston, Texas in the 2024–2025 timeframe with inconsistent, temporary, or no food access were more likely to report poor health and unemployment than those with reliable food access. Effect sizes ranged from small-to-moderate to moderate (φ = 0.33–0.36).

	A. Health status				
Food Access	Poor Health n (%)	OK/Excellent Health n (%)	χ^2^ (df)	p	Effect Size (φ)
Inconsistent/temporary/none (*n* = 20)	6 (30.00)	14 (70.00)	4.03 (1)	0.05	0.33
Reliable (*n* = 18)	1 (5.56)	17 (94.44)			

	**B. Food Access**				
**Employment Status**	**None n (%)**	**Part/Full-Time n (%)**	**χ**^**2**^**(df)**	**p**	**Effect Size (φ)**
Inconsistent/temporary/none (*n* = 21)	18 (85.71)	3 (14.29)	4.91 (1)	0.03	0.36
Reliable (*n* = 17)	9 (52.94)	8 (47.06)			

To further investigate what factors may be driving the ability to procure food, we found that employment status (e.g. no work vs. full- or part-time employment) showed a significant association with reliable food access (*p* = 0.03, chi-squared test; Table 2). Individuals with inconsistent, temporary, or no food access were substantially more likely to report having no employment (85.71%) than those with reliable food access (52.94%). Conversely, participants with reliable food access were more likely to report part-time or full-time employment (47.06%) than those with inconsistent food access (14.29%). The effect size was moderate (φ = 0.36).

Transportation access did not show an association with food access.

## 4 Discussion

In this study of people experiencing episodic homelessness in the greater Houston area, we found that food insecurity showed a significant association with health status. Additionally, employment status was significantly associated with reliable food access, while transportation and gross financial status did not.

Previous studies have found a similar association between food insecurity and health. More recently during the COVID-19 pandemic in the United States, a study of over 1.5 million adults found that food insufficiency showed a significant negative association with self-reported health status (Choi and Kim, 2024).

The association between employment with food access has also been demonstrated in previous literature. A study of over 14,000 people found a positive association between unemployment and food insecurity (Huang et al., 2016). A similar association was found in a previous study of 3494 supplemental nutrition assistance program (SNAP) unemployment and training (E&T) participants, which indicated that both loss of employment and reduction in earnings are associated with food insecurity (Mabli et al., 2023).

In contrast to our study, other studies have found associations between unreliable transportation access and food insecurity, with one study finding personal vehicle access reduced risk of food insecurity in public housing residents (Martinez et al., 2019). However, a Houston area study found poor walkability to be more often associated with areas of high food insecurity than poor public transit (Kakarala et al., 2023), potentially clarifying the lack of association between transportation barriers and food insecurity for this population. In addition, the small sample size of only eight individuals with access to reliable transportation may not be representative of the broader population of individuals experiencing episodic homelessness in the Houston area.

This study of the role of non-medical factors in the health of individuals experiencing episodic homelessness may ultimately lead to benefits in terms of policy and practice. For example, policymakers may be more likely to invest in preventive, upstream interventions if evidence demonstrates that non-medical factors contribute substantially to poor health outcomes. Additionally, in the practice of healthcare for this population, healthcare providers, social workers and community organizations may be better equipped to identify and address social factors that influence health outcomes. These findings may lead to more prevalent screening for these factors as a routine component of care.

Limitations of this study included challenges in subject recruitment, resulting in a smaller sample size of 40 compared to the target sample size of 61, and likely contributing to the lack of a significant association between food insecurity and health outcomes. The difficulty in studying people experiencing episodic homelessness is reflected in a broader paucity of research of this population, with a variety of factor contributing, including a lack of reliable contact information, and participant privacy concerns. Strengths of this study included the exploration of factors influencing health status in an understudied population. In addition, this study utilized both in-person and phone interviews to meet participant needs for ease of data collection, and we ensured confidentiality to our participants to help decrease fears surrounding divulging of sensitive personal information.

Future research may benefit from pooling together the data from populations of episodically homeless people across cities. Coalition-building between researchers of this difficult to study population may make it possible to analyze larger sample sizes.

## 5 Conclusion

In individuals experiencing episodic homelessness in the Houston area, reliable food access was found to have a significant association with self-reported health status. Having part- or full-time employment was significantly associated with reliable food access in this population, while transportation access and gross financial status were not.

## Studies in human

This study was performed in compliance with relevant laws, regulatory frameworks and guidelines where the research took place. This study was approved by the UTMB IRB (Approval No. 24-0053).

## CRediT authorship contribution statement

**Neki Yazdi:** Writing – review & editing, Writing – original draft. **Laura Porterfield:** Writing – review & editing, Writing – original draft, Methodology, Conceptualization. **Namita Bhardwaj:** Writing – review & editing, Writing – original draft, Methodology, Conceptualization. **Kendall M. Campbell:** Writing – review & editing, Methodology, Conceptualization. **Truong Lam:** Methodology, Investigation, Conceptualization. **Emily Rodgers:** Methodology, Data curation, Conceptualization. **Geraldo Hernandez:** Methodology, Data curation, Conceptualization. **Audrey Nath:** Writing – review & editing, Writing – original draft, Supervision, Methodology, Investigation, Formal analysis, Data curation, Conceptualization.

## Ethics declaration

### Informed consent and patient details

Written informed consent to take part in the study and to publish the article has been obtained from all participants or their legal representatives. The privacy rights of participants have been observed.

## Declaration of competing interest

The authors declare that they have no known competing financial interests or personal relationships that could have appeared to influence the work reported in this paper.

## Appendix A Supplementary data

Supplementary material Questionnaire items used to assess self-reported health status and selected social determinants of health, including food access, employment status and transportation reliability.
