# Supplementary material for: Social and environmental factors impacting the health in adults experiencing episodic homelessness in Houston, Texas
Source: Prev Med Rep. 2026 Oct 23;71:103681. doi: 10.1016/j.pmedr.2026.103681 (PMC13740878; doi:10.1016/j.pmedr.2026.103681)
Supplement: Supplementary file 1 [file mmc1.docx]

Supplemental Figure S1. Questionnaire items used to assess self-reported health status and selected social determinants of health, including food access, employment status, and transportation reliability.
